# Functional Near-Infrared Spectroscopy Recordings of Visuospatial Working Memory Processes. Part II: A Replication Study in Children on Sensitivity and Mental-Ability-Induced Differences in Functional Activation

**DOI:** 10.3390/brainsci8080152

**Published:** 2018-08-12

**Authors:** Joëlle S. Witmer, Eva A. Aeschlimann, Andreas J. Metz, Stefan J. Troche, Thomas H. Rammsayer

**Affiliations:** Institute of Psychology, University of Bern, 3012 Bern, Switzerland; joelle.witmer@psy.unibe.ch (J.S.W.); eva.aeschlimann@psy.unibe.ch (E.A.A.); andreas.j.metz@gmail.com (A.J.M.); stefan.troche@psy.unibe.ch (S.J.T.)

**Keywords:** fNIRS, visuospatial working memory processing, children, sensitivity, subjective task demand, mental ability, replication, generalization

## Abstract

In a previous study in young adults, we showed that hemodynamic changes as measured by functional near-infrared spectroscopy (fNIRS) were sensitive for identifying visuospatial working memory (WM)-related functional brain activation in the prefrontal cortex. This functional activation, however, could not be verified for participants with far-above-average mental ability, suggesting different cognitive processes adopted by this group. The present study was designed to confirm these findings in 11- to 13-year-old children by applying the same study design, experimental task, fNIRS setup, and statistical approach. We successfully replicated the earlier findings on sensitivity of fNIRS with regard to visuospatial WM-specific task demands in our children sample. Likewise, mental-ability-induced differences in functional activation were even more pronounced in the children compared with in the young adults. By testing a children sample, we were able to not only replicate our previous findings based on adult participants but also generalize the validity of these findings to children. This latter aspect seems to be of particular significance considering the relatively large number of fNIRS studies on WM performance in children.

## 1. Introduction

More than 20 years ago, the noninvasive and continuous measurement of brain activation with functional near-infrared spectroscopy (fNIRS) was introduced (e.g., [1,2]). A major advantage of fNIRS over other neuroimaging techniques such as positron emission tomography (PET) and functional magnetic resonance imaging (fMRI) is its compact measurement system [3] that puts less strain on participants. Moreover, it is less sensitive to motion artifacts [3], which enables its use in a variety of experimental settings [4]. With fNIRS, functional brain activation is measured noninvasively [5] by recording changes in oxygenated and deoxygenated hemoglobin (Hb) concentrations. The typical pattern of the hemodynamic response during functional brain activation is an increase in oxygenated hemoglobin (O_2_Hb) and a concomitant decrease in deoxygenated hemoglobin (HHb) [6]. As a consequence, the two signals are negatively correlated during functional activation [7,8,9]. Commonly, the concentration change of HHb is noticeably smaller than that of O_2_Hb [10,11].

Due to the advantages mentioned above, fNIRS has been increasingly used in cognitive research [12]. Several studies have assessed mental workload with verbal working memory (WM) tasks of varying load conditions and confirmed that increasing task difficulty resulted in increased O_2_Hb and decreased HHb concentrations [13,14,15,16,17]. The same pattern of Hb concentration changes could also be shown for visuospatial WM tasks (e.g., [18]). Validation studies using simultaneous fNIRS and fMRI measurements revealed a high correlation between the fNIRS-Hb and fMRI-blood oxygen-level dependent (BOLD) signals in motor tasks [19,20,21,22,23,24] as well as in WM tasks [22,25,26].

An alternative approach for validating the usefulness of fNIRS in WM research aims at providing evidence for the sensitivity of the fNIRS signal with regard to WM-specific task demands. More specifically, sensitivity refers to the question of whether the fNIRS technology is able to dissociate task-specific from more general task-unspecific processes. In order to pin down task-specific processes, an active control condition can be implemented where most of the perceptual and response-related processes are identical to those in the experimental condition whereas the task-specific processes of interest are only required for the experimental task. Differences in functional activation between the active control and experimental condition can then be attributed to the task-specific functional activation. This approach of validation, which is not based on a comparison with another method but on a variation of the task design, was realized in a first study by Witmer et al. [27]. In this study, the functional activation in Brodmann area (BA) 8 during the processing of a visuospatial WM task was investigated. BA 8, including the superior frontal gyrus, has been shown to be crucial for visuospatial WM processing (cf. Witmer et al. [27]). For example, in a lesion study by du Boisgueheneuc et al. [28], patients with lesions in the superior frontal gyrus, especially of the left hemisphere, showed deficits in visuospatial WM tasks. Similarly, a meta-analysis by Rottschy et al. [29] also confirmed that memory of stimulus location and the reproduction of a memorized stimulus lead to an increased functional activation in the left superior frontal gyrus.

Witmer et al.’s [27] sample consisted of young adults, 22 female and 21 male, ranging in age from 18 to 24 years. In the active control condition, there was no indication of a significant difference between O_2_Hb and HHb concentration changes. In the experimental condition, however, a statistically significant difference between O_2_Hb and HHb concentration changes occurred due to an increase in O_2_Hb and a simultaneous decrease in HHb concentration. This opposite effect on O_2_Hb and HHb concentration changes clearly corresponds to the typical hemodynamic response pattern observed during functional activation [6,30].

The primary goal of the present study was to investigate whether this effect was a one-time result or whether it can be replicated, and whether it occurs in other samples of participants. The necessity to replicate significant study results is strongly encouraged by the current debate referred to as the replication crisis (e.g., [31,32,33]). By applying the same study design, experimental task, fNIRS setup, and statistical approach as in our previous study [27], the current study conforms to a major requirement of good research practices derived from the replication crisis (e.g., [34]).

In order to critically evaluate the replicability and sample independence of Witmer et al.’s [27] results, a sample of children was examined in the present study. The fNIRS method is very well suited for measurements in children. Scalp–brain distances affect the photon path and have a direct impact on fNIRS data quality as indicated by the noise level in the data [26]. As the scalp–brain distance at the forehead is substantially shorter in children than in adults, measurements of children provide a better signal-to-noise ratio and, thus, a clearer fNIRS signal compared with adults [26,35,36].

Several studies pointed out that the pattern of functional activation during cognitive processing varies among individuals with different levels of mental ability. Early neuroimaging studies using single-photon emission computed tomography (SPECT) and PET reported less functional activation during the processing of cognitive tasks in individuals with higher mental ability compared with in individuals with lower mental ability (e.g., [37,38,39]). More recent fMRI studies provided converging evidence that this differential effect arises in particular when cognitive tasks with low to medium levels of task difficulty were used [40,41,42,43,44].

Such a mutual interference between level of mental ability and cognitive task demand on functional activation was recently confirmed by an fNIRS study using low- and high-conflict decision-making tasks [45]. While low-conflict decisions, involving lower cognitive task demands, produced a weaker hemodynamic response in the frontal cortex of participants with higher mental ability compared with in participants with lower mental ability, this pattern of functional activation did not occur in high-conflict decisions with enhanced cognitive task demands.

Overall, these studies suggested that individuals with lower mental ability (LA) show stronger functional activation compared with individuals with higher mental ability (HA) during the processing of a given cognitive task with low to moderate levels of task difficulty. At the same time, however, LA individuals performed more poorly than HA individuals [38,41,42,43,44,45]. This latter finding indicates that the same cognitive task with a low to moderate level of task difficulty was subjectively more demanding for LA than for HA individuals. Therefore, it appears reasonable to assume that the observed differences in functional brain activation were caused by the unequal subjective task demands experienced by LA and HA participants. These differences should thus disappear when participants perform a cognitive task controlled for the level of subjective task demand.

This issue was also addressed in Witmer et al.’s [27] study. In order to assess whether there is a moderating effect of mental ability on functional activation, participants were selected in a way that allowed assigning them to a LA or a HA group. Participants’ mental ability was assessed by Cattell’s Culture Fair Test 20-R [46]. To prevent an overlap in the 95% confidence interval between the two groups, only participants with IQ scores ranging from 90 to 112 (LA group) and from 130 to 145 (HA group) were included in the study. By applying an adaptive approach, Witmer et al. [27] ensured that all participants performed the same visuospatial WM task with task difficulty individually adjusted to obtain an equivalent level of subjective task demand irrespective of the participant’s individual level of mental ability. Hence, if the stronger functional activation in the LA group compared with in the HA group described in previous studies was due to higher subjective task demands, we would no longer expect differences, as the subjective task difficulty was comparable for both groups.

Despite the virtually identical subjective task demands for all participants in Witmer et al.’s [27] study, the difference in functional brain activation between the LA and HA group was still present. In the LA group, the WM-specific task demands of the experimental condition yielded the typical pattern of functional activation as indicated by an O_2_Hb increase and a concomitant HHb decrease. The HA group, on the contrary, displayed an almost identical pattern of Hb concentration changes in both the active control and experimental condition without any indication of functional activation. This finding clearly argues against the notion that the observed differences in functional brain activation originated from differences in subjective task demands. Rather, LA participants seemed to require more cortical oxygen compared with HA participants for solving an easy visuospatial WM task adjusted for subjective task demand.

Taken together, the major goal of the present study was to replicate the finding that fNIRS is sufficiently sensitive to measure visuospatial WM-specific processes. As an indication of sufficient sensitivity of fNIRS regarding visuospatial WM-specific processes, we predicted that the two-way interaction between Hb oxygenation and task condition would become significant. In particular, we expected a more pronounced functional activation, as indicated by the typical pattern of an increase in O_2_Hb and a simultaneous decrease in HHb, during the experimental condition compared with during the active control condition. The second goal of the present study was to reproduce the moderating effect of mental ability on functional activation. We expected significant differences in functional brain activation between the control and experimental condition in the LA but not in the HA group. In the LA group, the increase in O_2_Hb and the simultaneous decrease in HHb should be substantially more pronounced in the experimental compared to the active control condition. For the HA group, we did not expect any functional activation in either of the two task conditions. At the statistical level, this pattern should become evident from a significant three-way interaction between Hb oxygenation, task condition, and mental ability.

## 2. Materials and Methods

### 2.1. Participants

More than 250 children ranging in age from 11 to 13 years were recruited from public schools and screened for mental ability. In order to determine two mental ability groups whose 95% confidence intervals did not overlap, only children with an intelligence quotient (IQ) lower than or equal to 96 and higher than or equal to 115 were selected for the present study. This procedure resulted in a lower-ability (LA) group with IQ scores ranging from 72 to 96 (mean IQ score ± standard deviation: 89 ± 7.17) and a higher-ability (HA) group with IQ scores ranging from 115 to 141 (mean IQ score ± standard deviation: 122 ± 7.88). The LA group consisted of eight boys and ten girls (mean age: 11.6 ± 0.61 years), and the HA group consisted of 12 boys and 12 girls (mean age: 11.8 ± 0.59 years).

All participants were right-handed, and caregivers reported normal hearing and normal or corrected-to-normal vision. Based on a health questionnaire filled in by the caregiver of each child, all children were healthy with no history of psychiatric or neurological illness, serious head injury, or medication intake. The caregiver of each participating child provided written informed consent after being given detailed information on the study protocol and the NIRS recording procedure. In addition, the children gave oral consent. The study was conducted in accordance with the Declaration of Helsinki and the study protocol was approved by the ethics committee of the Faculty of Human Sciences of the University of Bern (Bern, Switzerland) (date of approval: 29 July 2014; project identification code: No. 2014-6-880651).

### 2.2. Assessment of Psychometric Intelligence

Mental ability was assessed by the short version of the German adaptation of Cattell’s Culture Fair Intelligence Test (CFT 20-R; [46]). The CFT 20-R is a nonverbal test that consists of four different inductive reasoning subtests and can be considered an estimate of an individual’s general fluid intelligence [46,47]. The time allowed was 4 min for Subscales 1 and 2 and 3 min for Subscales 3 and 4. As a measure of mental ability, the number of correctly solved items was transformed into an IQ score. The CFT 20-R manual provides IQ norms for test takers ranging in age from 8.5 to 60 years.

### 2.3. Quantification of Individual Visuospatial WM Spans

A combined version of the Patterns–Memory Task [48] and the Matrix Task [49] was used to quantify individual visuospatial WM spans. The task was computer-controlled and programmed with E-Prime experimental software (Psychology Software Tools, Inc., Sharpsburg, PA, USA). Each trial started with the presentation of a fixation cross for 500 ms, followed by a black 4 × 4 grid (13.2 × 13.2 cm) consisting of 16 white squares, referred to as the empty grid, presented in the center of a touchscreen monitor. The viewing distance was about 60 cm and sufficiently close to the child to comfortably reach the touchscreen monitor without body movement.

The task started with a block of four trials with a set of two squares to be memorized. More precisely, two of the 16 squares successively turned black for 1000 ms. Participants were instructed to memorize the order of appearance and the location of the black squares. Next, a question mark was displayed for 1000 ms, followed by the empty grid that was used as an input template for the participant’s response. The participant was prompted to touch, in reverse order, those two squares on the empty grid that previously turned black. The second block comprised four trials with a set of three squares to be memorized, the third block comprised four trials with a set of four squares to be memorized, and so on. Thus, if the participant was able to correctly solve at least three trials within a given block, the set of squares to be memorized was extended by one additional square in the next block. The number of squares of the last block in which this criterion had been met constituted the participant’s visuospatial WM span. The black squares were presented in a pseudo-randomized order in all trials.

### 2.4. Experimental and Active Control Conditions of the fNIRS Study

The procedure and stimuli were identical to those in the ordinary WM task, with the exception that no adaptive procedure was applied. For the fNIRS recordings, an experimental condition, i.e., the visuospatial WM task, was contrasted with an active control condition. This enabled the application of the subtraction approach, which allows the identification of WM-specific changes in Hb oxygenation concentration [6,50,51].

In the experimental condition, visuospatial WM-specific processes as well as WM-unspecific processes, such as perceptual encoding of the stimuli, response selection, and execution of the motor response, were required. For each child, an individual level of task difficulty was chosen based on his/her WM span determined in the previous experimental session. For example, a child with an individual WM span of four items was presented with a series of four black squares that appeared successively in a pseudo-randomized order. The child was instructed to memorize the order of appearance and the location of the four black squares and subsequently prompted to touch, in reverse order, those four squares on the empty grid that previously turned black. With this procedure, subjective task demand was held constant for all children.

In the active control condition, the number of squares that turned black was identical to the experimental condition. Unlike in the experimental condition, however, the squares no longer turned black in a pseudo-randomized but in a systematic order (either from left to right or from top to bottom). As a result, the task demand on WM was extensively reduced in the active control compared to the experimental condition, whereas the task-unspecific processes were identical in both conditions.

**Figure 1:**
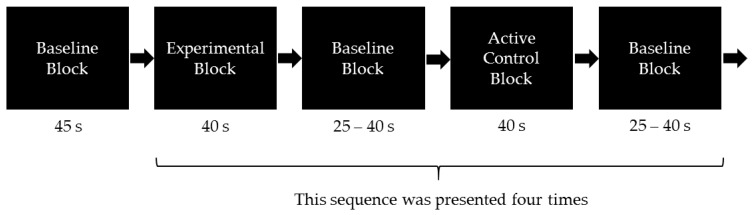
Illustration of the block design, which contained eight baseline blocks (resting phases), four experimental blocks, and four active control blocks. After the first baseline block, participants started either with an experimental or an active control block (order was counterbalanced across participants).

Task instructions were given orally via headphones. Prior to the proper experiment, each child completed seven practice trials to refresh the WM task and to ensure that the task instructions were fully understood. The fNIRS measurement started with a 45 s resting phase, followed by the task conditions. The entire fNIRS visuospatial WM task consisted of four blocks of the active control condition and four blocks of the experimental condition. Both types of blocks were presented for 40 s each in alternating order. The order of the experimental and active control blocks was counterbalanced across participants. All blocks were separated from each other by a resting phase referred to as baseline block (see Figure 1). During the resting phase, children were instructed to sit quietly with their eyes open. To reduce the influence of Mayer waves, which are spontaneous oscillations in blood flow that affect the fNIRS signal [52], and to prevent influences of blood flow changes due to rhythmic changes, the duration of the resting phases was randomly varied from 25 to 40 s. As a behavioral measure, the respective percentages of correctly solved trials were determined for the experimental and the active control condition.

### 2.5. fNIRS Recordings

**Figure 2:**
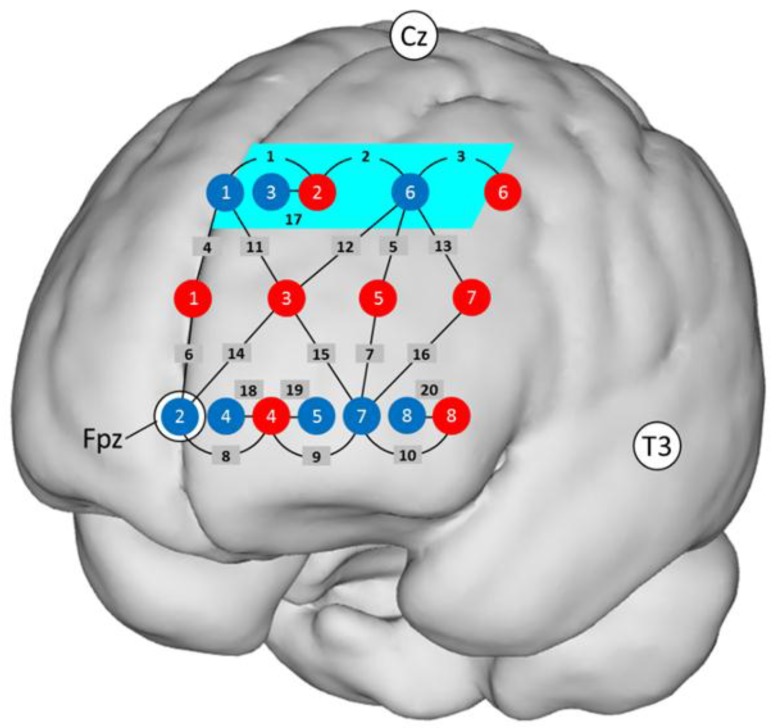
Localization of the 20 channels projected on a standard brain. There were eight detectors (blue dots) and eight emitters (red dots). The black numbers indicate channels. The area highlighted in turquoise (Channels 1, 2, and 3) corresponds to the region of interest (ROI). Channels 1–10 have a source–detector distance of ~30 mm; Channels 11–16, ~42 mm; Channels 17–20, ~15 mm. Detector 2 was placed at Fpz, Source 1 and Detector 1 on an imaginary line towards Cz, and the bottom row of fibers on an imaginary line towards T3.

Functional near-infrared spectroscopy (fNIRS) data were acquired and preprocessed exactly the same way as in Witmer et al.’s [27] study. Briefly summarized, a multichannel system (FOIRE 3000, Shimadzu Corporation, Kyoto, Japan) was used which recorded optical density changes from 20 channels (light source–light detector combinations) at three wavelengths (780 nm, 805 nm, 830 nm) and at three different distances (four channels at ~15 mm, 10 channels at ~30 mm, six channels at ~42 mm). The optical probe included an array of 16 light fibers connected to the fNIRS system (eight sources and eight detectors). The probe holder was placed over the left frontal scalp, between the 10–20 landmarks Fpz, Cz, and T3 (see Figure 2). Every light fiber position was measured in Montreal Neurological Institute (MNI) coordinates using a FASTRAK^®^ 3D magnetic field digitizer (Polhemus, Colchester, VT, USA). O_2_Hb and HHb relative concentration changes were calculated from the optical densities. The short channels (~15 mm) were used to regress out superficial influences [4,6] from the longer channels [53], but only if the correlation between the time series of the two signals was sufficient, such that R^2^ > 0.1 [54].

To remove movement artefacts, a movement artifact removal algorithm (MARA; [55]) was applied. After that, O_2_Hb and HHb concentration changes were calculated across the four experimental and the four control blocks, respectively, by means of a general linear model (GLM) model as suggested by Gagnon et al. [56]. All children achieved at least 80% and 50% correct responses in the control and experimental condition, respectively. For statistical analysis, the mean concentration changes in O_2_Hb and HHb relative to baseline were calculated within each child for both conditions (control and experimental) and for each channel (Channels 1–16).

### 2.6. Time Course of the Study

A three-stage procedure was used in the present study. First, over 250 children were screened for mental ability. Then, those children who qualified for either the LA or HA mental ability group performed an adaptive visuospatial WM task to determine the required individual level of task demand for the subsequent fNIRS measurement. The average interval between the three testing stages was 18 days.

## 3. Results

All statistical analyses were run with R [57]. The post hoc pairwise comparisons were adjusted for multiple testing with Bonferroni–Holm correction [58].

### 3.1. Behavioral Data

The mean WM span (± standard deviation) of the total sample was 4.0 ± 0.98. For the LA and the HA groups, mean WM spans were 3.8 ± 1.00 and 4.3 ± 0.94, respectively. When performing the individually adjusted WM task during fNIRS measurement, the mean percentages of correct responses across all participants were 99.5 ± 1.3% and 92.1 ± 6.1% for the active control and the experimental condition, respectively, *t*(41) = 7.88, *p* < 0.001, *d* = 1.67. This finding clearly shows that the task demands were significantly higher in the experimental than in the active control condition. At the same time, percentages of correct responses did not differ significantly between the LA and HA group in the experimental condition; mean percentages of correct responses were 91.4 ± 6.2% and 92.6 ± 6.2% for the LA and the HA group, respectively, *t*(40) = 0.62, *p* = 0.54, *d* = 0.19. Thus, we accomplished our goal of obtaining equivalent levels of subjective task difficulty for both mental ability groups.

### 3.2. fNIRS Data

Definition of the region of interest (ROI): In order to enable a best possible replication of Witmer et al.’s [27] findings, we investigated the same ROI, located in BA 8, and used the same exploratory approach. This ROI was represented by the Channels 1 to 3. The MNI coordinates (x, y, z) of Channels 1 to 3 were (−8, 48, 48), (−27, 40, 48), and (−42, 25, 48), respectively. In all three channels a negative correlation between O_2_Hb and HHb could be observed, caused by an increase in O_2_Hb and a decrease in HHb. This typical hemodynamic response pattern during functional activation [6] was a good indication that the signal did not contain systemic changes [6,59] and demonstrated high signal quality [8]. The mean concentration changes in O_2_Hb and HHb of Channels 1 to 3 were averaged and, thus, provided the value for our ROI. Within this ROI, the negative correlations between O_2_Hb and HHb were significant for both conditions (*r*_control_ = −0.46, *r*_experimental_ = −0.53 (both *p* values <0.01, two-tailed)). Figure 2 shows a projection of the ROI onto a standard brain. Obtained concentration changes in O_2_Hb and HHb as a function of fNIRS condition are presented in Table 1, for the total sample as well as for the LA and the HA groups.

Next, we assessed our two main hypotheses derived from Witmer et al.’s [27] study. First, changes in functional activation while performing the WM task were expected to be specific to visuospatial WM-related processes and not related to more general subsidiary processes. Second, the individual level of mental ability was predicted to effectively moderate the concentration changes in Hb oxygenation during the processing of the visuospatial WM task. More precisely, substantial functional activation was expected for the LA but not for the HA group. To verify our hypotheses, a three-way analysis of variance was performed with Hb oxygenation (O_2_Hb and HHb) and task condition (experimental and control) as the two within-subject factors and mental ability (LA and HA) as a between-subjects factor.

This analysis yielded a significant main effect of Hb oxygenation, *F*(1, 40) = 13.76, *p* < 0.001, *η_p_*^2^ = 0.256, indicating a much more pronounced change in O_2_Hb than HHb concentration. Neither the main effect of task condition, *F*(1, 40) = 2.24, *p* = 0.142, *η_p_*^2^ = 0.053, nor the main effect of mental ability, *F*(1, 40) = 0.02, *p* = 0.892, *η_p_*^2^ < 0.001, reached the 5% level of statistical significance. There was, however, a statistically significant two-way interaction of Hb oxygenation and task condition, *F*(1, 40) = 6.38, *p* = 0.016, *η_p_*^2^ = 0.138, as well as a statistically significant three-way interaction of all factors combined, *F*(1, 40) = 4.24, *p* = 0.046, *η_p_*^2^ = 0.096. No significant two-way interactions were found for Hb oxygenation and mental ability, *F*(1, 40) = 0.27, *p* = 0.601, *η_p_*^2^ = 0.007, or for task condition and mental ability, *F*(1, 40) = 3.63, *p* = 0.064, *η_p_*^2^ = 0.083.

**Figure 3:**
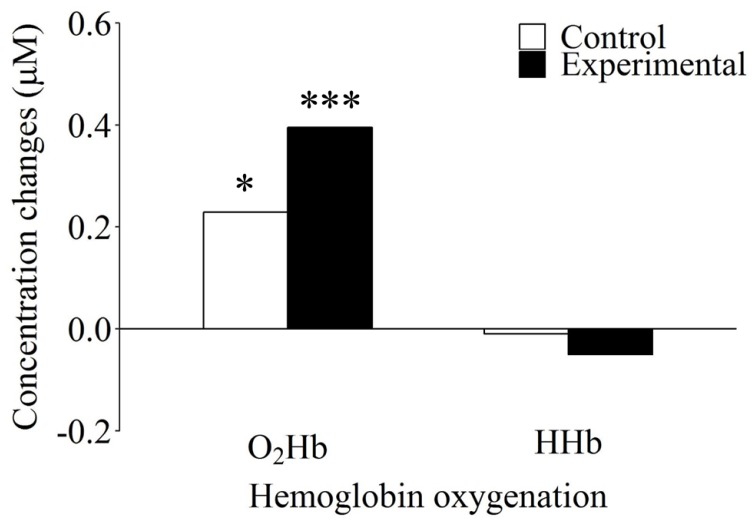
Differences in concentration changes for oxygenated (O_2_Hb) and deoxygenated (HHb) hemoglobin as a function of condition for the working memory task. Within the control and the experimental condition, there was a significant difference in concentration change between O_2_Hb and HHb. *: significantly different from HHb concentration change in the active control condition (*p* < 0.05). ***: significantly different from HHb concentration change in the experimental condition (*p* < 0.001).

With regard to the significant two-way interaction of Hb oxygenation and task condition, post hoc pairwise comparisons revealed that the O_2_Hb concentration was significantly higher than the HHb concentration in the active control condition, *t*(41) = 2.28, *p* = 0.028, *d* = 0.57. Furthermore, in the experimental condition, an increase in O_2_Hb and a concomitant slight decrease in HHb resulted in a highly significant difference between the O_2_Hb and the HHb concentrations, *t*(41) = 4.26, *p* < 0.001, *d* = 1.09 (see Figure 3). This pattern of changes in O_2_Hb and HHb concentrations is consistent with the notion that the observed functional brain activation can be considered specific to visuospatial WM-related processes rather than reflecting more general subsidiary processes.

**Figure 4:**
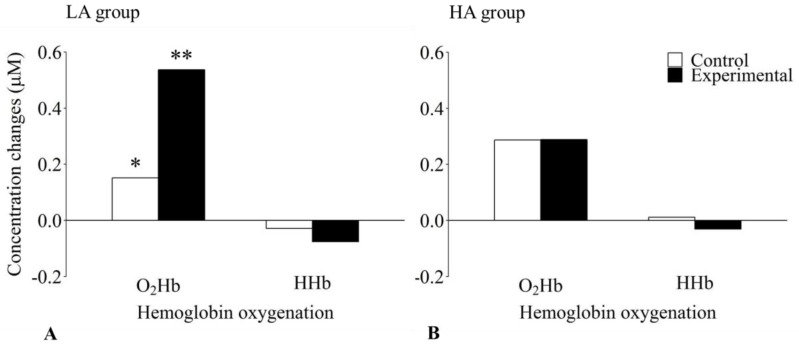
Effects of level of mental ability and condition on hemoglobin oxygenation concentration changes for the working memory task in the lower (LA; Panel **A**) and the higher (HA; Panel **B**) mental ability group. The lower mental ability group showed significantly increased oxygenated hemoglobin (O_2_Hb) and significantly decreased deoxygenated hemoglobin (HHb) concentrations in the experimental compared to the active control condition. No significant differences could be observed for the higher mental ability group. *: significantly different from O_2_Hb concentration change in the experimental condition (*p* < 0.05). **: significantly different from HHb concentration change in the experimental condition (*p* < 0.01).

Regarding the statistically significant three-way interaction, it should be noted that the occurrence of functional brain activation might be differentially affected by an individual’s level of mental ability. To assess the interaction more thoroughly, two separate two-way analyses of variance were conducted with the within-subject factors Hb oxygenation and task condition for the LA and the HA groups, respectively. For the LA group, a statistically significant main effect of Hb oxygenation, *F*(1, 17) = 7.12, *p* = 0.016, *η_p_*^2^ = 0.295, indicated a higher O_2_Hb concentration compared with that of HHb. No significant main effect of task condition, *F*(1, 17) = 2.79, *p* = 0.113, *η_p_*^2^ = 0.141, was revealed. Most importantly, however, the interaction between these two factors became also significant, *F*(1, 17) = 7.40, *p* = 0.015, *η_p_*^2^ = 0.303. Post hoc pairwise comparisons indicated that the O_2_Hb concentration was significantly higher in the experimental than in the active control condition, *t*(17) = 2.26, *p* = 0.037, *d* = 0.66. Unlike O_2_Hb, no significant difference in HHb concentration between the experimental and the active control condition could be revealed, *t*(17) = 0.74, *p* = 0.235, *d* = 0.19. For the active control condition, no indication of a significant difference between O_2_Hb and HHb concentration was found, *t*(17) = 1.07, *p* = 0.198, *d* = 0.39. In the experimental condition, however, there was a significant difference between O_2_Hb and HHb due to the increase in O_2_Hb and a slight concomitant decrease in HHb concentration, *t*(17) = 3.62, *p* = 0.004, *d* = 1.41 (see left panel of Figure 4).

As in the LA group, a two-way analysis of variance for the HA group yielded a statistically significant main effect of Hb oxygenation, *F*(1, 23) = 6.40, *p* = 0.019, *η_p_*^2^ = 0.218, but no statistically significant main effect of task condition, *F*(1, 23) = 0.24, *p* = 0.627, *η_p_*^2^ = 0.010. In contrast to the LA group, there was no indication of a statistically significant interaction between Hb oxygenation and task condition, *F*(1, 23) = 0.16, *p* = 0.698, *η_p_*^2^ = 0.007 for the HA group (see right panel of Figure 4). Thus, the additional analysis of the statistically significant three-way interaction confirmed functional brain activation elicited by visuospatial WM-specific processes in the participants with lower mental ability, whereas in the participants with higher mental ability no evidence of functional activation could be identified.

## 4. Discussion

The major aim of the present study was to replicate two results of the study by Witmer et al. [27]: (1) the sensitivity of fNIRS recordings for measuring visuospatial WM-specific processes and (2) the moderating effect of mental ability on functional activation. An additional goal was to test whether these results can be generalized to a children sample.

Because fNIRS is markedly less burdensome for subjects than other neuroimaging procedures (e.g., fMRI or PET), it is often used for WM studies in children (e.g., [60,61,62]). For this reason, the generalizability of Witmer et al.’s [27] results to a children sample is important from a use-inspired perspective. Furthermore, due to children’s shorter scalp–brain distance at the forehead [63], fNIRS recordings in children provide a better signal-to-noise ratio and, thus, a clearer signal than recordings in adult subjects [26,35,36,64]. Hence, from a methodological perspective, fNIRS recordings in children, from which possible movement artifacts were carefully removed, should yield an enhanced signal quality compared with the adult sample investigated by Witmer et al. [27].

The pronounced functional activation elicited by visuospatial WM-specific task demands in the experimental compared with the active control condition, as observed in the present study, clearly confirmed Witmer et al.’s [27] finding. These concordant results provide converging evidence that fNIRS recordings are sensitive enough to reflect visuospatial WM-specific processes in both children and young adults.

The replication of the moderating effect of mental ability on functional activation was also successful in the children sample. In the LA group, the visuospatial WM-specific task demands of the experimental condition induced the typical pattern of functional activation, whereas in the active control condition no functional activation took place. In contrast, the HA group showed no indication of functional activation in either condition. This consistency with the outcome of Witmer et al.’s [27] study indicates that the differential effect of mental ability on functional brain activation is a fundamental phenomenon that not only occurs in adults but can also be detected in the developing brain of children.

A similar differential pattern of functional activation as a function of mental ability in adult subjects has been reported in previous PET studies [38,39], SPECT studies [37], fMRI studies [40,41,42,43,44], and an fNIRS study [45]. All these studies revealed that LA individuals show much more pronounced functional activation than HA individuals when processing cognitive tasks with low to moderate levels of task difficulty. It is important to note, however, that HA individuals consistently performed better than LA individuals when processing the same WM task. This latter finding clearly indicates that a given cognitive task with low to moderate difficulty is subjectively less demanding for HA compared to LA individuals. Therefore, it cannot be ruled out that this inequality in subjective task demand may have caused the observed differences in brain activation between LA and HA individuals.

To control for this crucial issue, we applied the same adaptive approach as Witmer et al. [27]. By adopting this approach, we ensured that each child was presented with a task that was individually adjusted for difficulty, to obtain a virtually identical level of subjective task demand irrespective of the child’s individual level of mental ability. At the behavioral level, this was confirmed by the fact that the percentages of correct responses in the experimental condition did not differ significantly between the LA and HA group. Despite the virtually identical subjective task demand for all children, the difference in functional brain activation between the LA and HA children was still present. This provides clear evidence against the notion that the observed differences in functional brain activation originate from differences in subjective task demand. Rather, LA children seemed to require more cortical oxygen compared with HA children for solving the visuospatial WM task adjusted for subjective task demand.

A comparison of the children’s data in the present study with the data of the young adults from the study by Witmer et al. [27] revealed that measured hemodynamic responses and, in particular, changes in O_2_Hb were markedly larger in the children sample by a factor of nearly four for both the experimental and the active control condition. Mean changes in O_2_Hb were 0.05 and 0.23 in the adult and children sample, respectively, in the control condition. Similarly, in the experimental condition, mean O_2_Hb changes were 0.14 and 0.40 for the adult and children sample, respectively. Such a difference in activation strength of the frontal lobe between children and young adults has also been reported in previous fMRI (e.g., [65,66,67]) and fNIRS [62] studies. The common aspect of these studies was that although they found activation in the same frontal brain areas for children and adults during processing of the same task, the activation was markedly stronger in children than in adults.

For fNIRS studies, the shorter scalp–brain distance in children provides the most reasonable explanation for this observation [35]. Cui et al. [26] and Strangman et al. [36] demonstrated that scalp–brain distances affect the photon path and have a direct impact on fNIRS data quality as indicated by the signal-to-noise ratio. As the scalp–brain distance at the forehead is substantially shorter in children than in adults, the signal-to-noise ratio of fNIRS recordings in children seems to be much better than that of adult subjects.

This aspect is also relevant for the interpretation of the differential effect of mental ability on functional activation in the present study. In the LA group, the expected functional brain activation pattern became even clearer (compared with adults), whereas in the HA group, there were no signs of functional activation despite the improved signal quality. Thus, the absence of functional brain activation in the HA group cannot be accounted for by a methodological artifact due to blurred signal, but represents a genuine null result [68].

One major methodological challenge of fNIRS technology is the fact that the NIRS signal reflects not only the genuine cortical hemodynamic response evoked by the experimental task, but also extracerebral hemodynamic effects [6,69,70]. These latter effects can mimic or mask the genuine cortical hemodynamic response [6]. To cope with this problem, a multidistance approach (e.g., [25,71,72,73]) was applied in the present study. This procedure enabled us to use the short channels to regress out superficial influences from the longer channels [52].

Another methodological issue of the present study is age- or maturation-related differences in the head size of children [74]. Substantial deviations may have resulted in channel locations that did not correspond to our specified ROI. For this reason, we used a 3D digitizer to measure the locations of the optodes and calculate the locations of the channels as described in Singh, Okamoto, Dan, Jurcak, and Dan [75]. This approach allowed for the identification of children whose channel locations did not correspond to our specified ROI.

## 5. Conclusions

Taken together, we successfully replicated two major findings reported by Witmer et al. [27]. By confirming that fNIRS is sensitive enough to assess hemodynamic responses directly related to cognitive processes elicited by a visuospatial WM task, we provide additional converging evidence for the validity and sensitivity of fNIRS recordings. Furthermore, we were able to replicate the mental-ability-induced differences in functional activation. Moreover, by testing a children sample, we were also able to generalize the validity of Witmer et al.’s [27] findings in young adults to 11- to 13-year-old children. This latter aspect seems to be of particular significance considering the relatively large number of fNIRS studies on WM performance in children.

## Figures and Tables

**Table 1:** Mean (*M*) and standard deviation (*SD*) of oxygenated (O_2_Hb) and deoxygenated (HHb) hemoglobin concentration changes as a function of functional near-infrared spectroscopy (NIRS) condition (control and experimental) for the total sample (Total, *N* = 42) as well as for the lower (LA, *n* = 18) and higher (HA, *n* = 24) mental ability groups.

Hemoglobin Oxygenation	Condition	Total		LA		HA	
		*M*	*SD*	*M*	*SD*	*M*	*SD*
O_2_Hb	control	0.23	0.54	0.15	0.59	0.29	0.49
	experimental	0.40	0.53	0.54	0.57	0.29	0.48
HHb	control	−0.01	0.22	−0.03	0.25	0.01	0.19
	experimental	−0.05	0.23	−0.08	0.23	−0.03	0.23
